# Genome sequence of *Escherichia coli* NCCP15653, a group D strain isolated from a diarrhea patient

**DOI:** 10.1186/s13099-016-0084-6

**Published:** 2016-02-23

**Authors:** Min-Jung Kwak, Myung-Soo Kim, Soon-Kyeong Kwon, Seung-Hak Cho, Jihyun F. Kim

**Affiliations:** Department of Systems Biology and Division of Life Sciences, Yonsei University, 50 Yonsei-ro, Seodaemun-gu, Seoul, 120-749 Republic of Korea; Division of Enteric Diseases, Center for Infectious Diseases, Korea National Institute of Health, Heungdeok-Gu, Cheongju, 363-951 Republic of Korea

**Keywords:** Extraintestinal *E. coli*, UPEC, NMEC, Cystitis, Pyelonephritis

## Abstract

**Background:**

Pathogenic strains in *Escherichia coli* can be divided into several pathotypes according to their virulence features. Among them, uropathogenic *E. coli* causes most of the urinary tract infections and has a genotype distinct from other virulent strains of *E. coli*. In this study, we sequenced and analyzed the genome of *E. coli* NCCP15653 isolated from the feces of a diarrhea patient in 2007 in South Korea.

**Results:**

A phylogenetic tree based on MLST showed that NCCP15653 belongs to the D group of *E. coli* and located in the lineage containing strains ST2747, UMN026 and 042. In the genome of NCCP15653, genes encoding major virulence factors of uropathogenic *E. coli* were detected. They include type I fimbriae, hemin uptake proteins, iron/manganese transport proteins, yersiniabactin siderophore proteins, type VI secretion proteins, and hemolysin. On the other hand, genes encoding AslA, OmpA, and the K1 capsule, which are virulence factors associated with invasion of neonatal meningitis-causing *E. coli*, were also present, while a gene encoding CNF-1 protein, which is a cytotoxic necrotizing factor 1, was not detected.

**Conclusions:**

Through the genome analysis of NCCP15653, we report an example of a genome of chimeric pathogenic properties. The gene content of NCCP15653, a group D strain, demonstrates that it could be both uropathogenic *E. coli* and neonatal meningitis-causing *E. coli*. Our results suggest the dynamic nature of plastic genomes in pathogenic strains of *E. coli*.

## Background

*Escherichia coli* can be divided into commensal and pathogenic strains. Commensal *E. coli* is a member of the normal flora of animal intestine and other body sites, but pathogenic strains of *E. coli* cause several health problems. Many *E. coli* strains can cause diarrhea, but not serious [1]. However, some pathogenic stains such as *E. coli* O104:H4 that caused the German outbreak in 2011 may be fatal [2]. According to the virulence factors and phenotypes, pathogenic *E. coli* strains can be classified into enteroaggregative *E. coli* (EAEC), enterohemorrhagic *E. coli* (EHEC), enteroinvasive *E. coli* (EIEC), enteropathogenic *E. coli* (EPEC), enterotoxigenic *E. coli* (ETEC), uropathogenic *E. coli* (UPEC), and *E. coli* that causes neonatal meningitis (NMEC) [3–8]. Among them, UPEC and NMEC are extraintestinal pathogenic *E. coli* (ExPEC), and most of the urinary tract infections (UTIs) are caused by UPEC strains [9]. The urinary tract is a harsh environment because of continuous urine excretion, antibacterial factors, and strong immune system, and these features of urinary tract can make UPEC possible to have genotypes distinct to other pathogenic strains [10]. In the urinary tract, it needs adhesion to urinary epithelial cells, several resistance factors against the antibacterial factors and host immune systems, and iron-acquisition systems to obtain iron, which is limited in the urinary tract. UPEC causes the infection in the bladder and sometimes in the kidneys through entering the ureters from the bladder to trigger symptoms such as cystitis and pyelonephritis, and even bacteremia and sepsis through entering the bloodstream [11]. In this study, we sequenced and analyzed the genome of pathogenic *E.coli* strain NCCP15653 isolated from the feces of a patient suffering from diarrhea.

## Methods

### Bacteria and DNA isolation

*E. coli* strain NCCP15653 was isolated from the feces of a Korean patient with the diarrhea symptom in 2007. This strain was deposited at the National Culture Collection for Pathogens in Korea National Institute of Health (KNIH) and its accession number is NCCP15653. Genomic DNA was extracted using chemical and enzymatic methods as described in Molecular cloning, a laboratory manual [12].

### Genome sequencing, de novo assembly and annotation

For the genome sequencing of NCCP15653, Genome Analyzer IIx of the Illumina platform at the Biomedical Genomics Research Center of the Korea Research Institute of Bioscience and Biotechnology was used and 18,521,148 of raw sequencing reads with 76-bp of average read length were generated from a 500-bp paired-end library. The sequencing reads were imported into CLC Genomics Workbench version 5.1 (CLC bio, Qiagen, Netherlands) with the parameters of 400–700 of paired-end distance and 1.5–1.7 version of Illumina quality score. Trimming of the imported reads was performed with the parameters of 0.01 quality score, none of the ambiguous nucleotide, and 70-bp of minimum read length. De novo assembly of 13,864,337 high-quality reads were conducted using CLC Genomics Workbench with the parameters of similarity fraction of 1.0, length fraction of 0.5, and minimum contig length of 500 bp. SSPACE [13] was used for scaffolding and IMAGE [14] was used for automatic gap filling. Manual contig extension and gap filling were performed with CLC Genomics Workbench. Structural gene prediction was accomplished with Glimmer3 [15], and functional annotation of predicted genes was performed using the MicroScope database [16].

### Genome analysis

A phylogenetic tree based on multilocus sequence typing (MLST) was constructed with MEGA5 [17]. Nucleotide sequences of seven MLST genes (*adk* adenylate kinase, *fumC* fumarate hydratase, *gyrB* DNA gyrase, *icd* isocitrate/isopropylmalate dehydrogenase, *mdh* malate dehydrogenase, *purA* adenylosuccinate dehydrogenase, *recA* ATP/GTP binding motif) [18] and Jukes-Cantor model were used for tree construction. To determine the serotype of NCCP15653 in silico, amino-acid sequences of the *wzx* and *wzy* gene for O-antigen and the *fliC* gene for H-antigen were used and the neighbor-joining trees were constructed with MEGA5. SerotypeFinder program [19] was also used for the analysis. Average nucleotide identity based on blast (ANIb) value was calculated using JSpecies [20]. Calculation of the core genome was conducted with OrthoMCL (ver. 2.0.3) [21] with parameters of *e*-value ≤1*e*–5, identity ≥85 %, and coverage ≥80 % [22]. Functional classification of the genes was conducted by BLASTP with the COG and subsystem databases. Prediction of phage sequences and clustered regularly interspaced short palindromic repeats (CRISPRs) was performed with PHAST [23] and CRISPRfinder [24], respectively. Detection of the virulence genes was conducted using BLAST software. Typing of the specific virulence genes were referenced to the virulence factors of pathogenic bacteria database (http://www.mgc.ac.cn/VFs/main.htm) [25].

## Quality assurance

*E. coli* NCCP15653 was maintained in pure culture at KNIH and genomic DNA was isolated from a single isolate. Possibilities for the contamination of other genomes and misassembly were checked through mapping reads to the contigs. The read mapping of the draft genome of NCCP15653 indicated that the distance between paired-end reads is in the range of expected size distribution and the coverage of the reads was consistent throughout the genome.

## Results and discussion

### General features

**Fig. 1:**
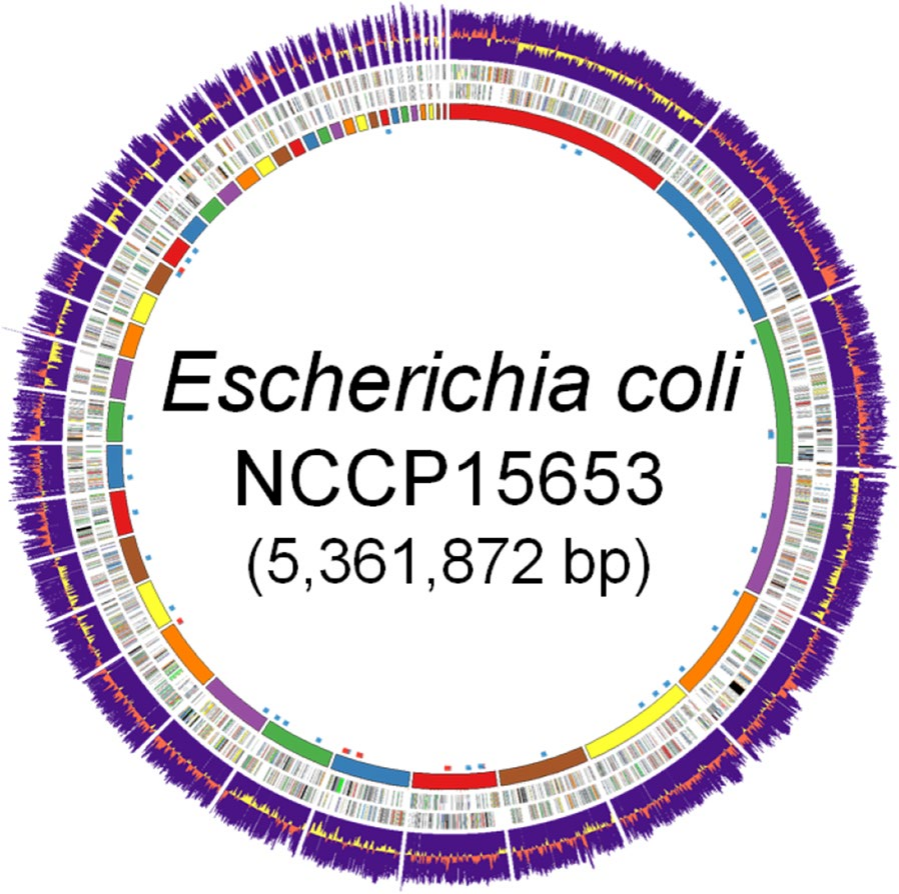
Circular representation of draft genome of NCCP15653. Circular map of the draft genome was constructed using the Circos program [37]. The *first* *purple circle* from outside is the GC content and *next circle* colored by *red* and *yellow* in the *first circle* indicates the GC skew. Next *two circles* are the color-coded genes according to the COG category. The *most inner circle* indicates the 43 contigs arranged in order to the size. *Blue and red scattered spots* are tRNAs and rRNA, respectively

The draft genome of *E. coli* NCCP15653 consists of 43 contigs and the sum of the length of the contigs is 5,361,872 bp with 50.56 % of GC content (Table 1 and Fig. 1). The number of predicted protein coding sequences (CDSs) is 5203 and the percentages of subsystem and COG assigned proteins were 76.40 and 76.42 % respectively. The numbers of predicted transfer RNA and ribosomal RNA are 73 and 21, respectively. In the genome of NCCP15653, six intact phages and four CRISPR candidates were detected. Among the four CRISPR candidates, one has the *cas* genes next to the repeat array and nine spacers.

**Table 1:** General features of the *E. coli* NCCP15653 genome

Item	Value
Number of contigs	43
Total contig length (bp)	5,361,872
Fold coverage (x)	171.19
N50 (bp)	242,877
G + C content (%)	50.56
Number of protein coding genes	5297
Number of predicted transfer RNAs	73
Number of predicted ribosomal RNAs	21
GenBank accession number	ATLY00000000

### Phylogenetic relationships

**Fig. 2:**
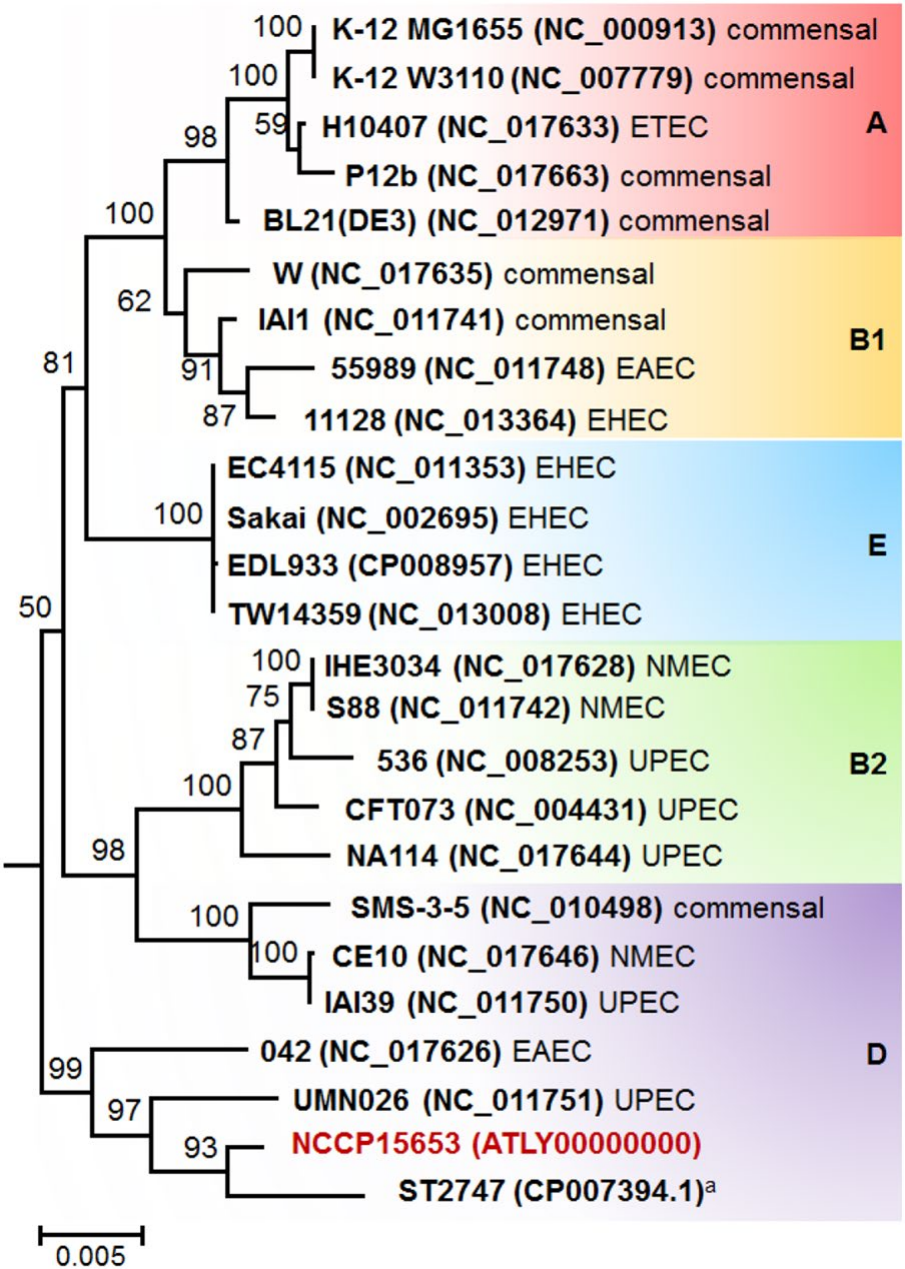
Phylogenetic relationships of *E. coli* strains. A phylogenetic tree based on maximum likelihood method was generated by MEGA5 with nucleotide sequences of seven MLST genes. Bootstrap values (percentages of 1000 replications) greater than 50 % are shown at each node. The scale bar represents 0.01 nucleotide substitutions per site. *E. fergusonii* ATCC 35469 was used for the out-group. Each color indicates the phylogenetic groups of *E. coli* (*red* A; *yellow* B1; *blue* E; *purple* D; *green* B2). ^a^ A strain isolated from the feces of patient with UTI [28], but its pathotype is yet to be identified

The phylogenetic tree based on MLST showed that NCCP15653 belong to the D group of *E. coli* (Fig. 2). In accordance with previous reports [26, 27], strains belonging to group D are contained in two distinct phylogenetic lineages and may have a polyphyletic origin. One is located in the outermost branches of *E. coli* outside the groups A, B1, B2, and E, and the other forms a sister clade of group B2. NCCP15653 is placed in the former clade. The *E. coli* group D includes several pathogenic strains such as ST2747 (isolated from feces of patient with UTI, but pathotype not identified) [28], UMN026 (UPEC), 042 (EAEC), IAI39 (UPEC), and CE10 (NMEC) as well as commensal strains SMS-3-5 [29–32]. NCCP15653 is placed next to strain ST2747. Calculation of ANIb between the group D strains also indicated that NCCP15653 is most similar to ST2747; average ANIb value and genome coverage are 98.18 and 85.54 %, respectively (Table 2). A serotype analysis using the genes encoding O-antigen and H-antigen indicated that O-antigen of NCCP15653 is untypable but H-antigen can be clustered with those of the H18 serotype.

**Table 2:**
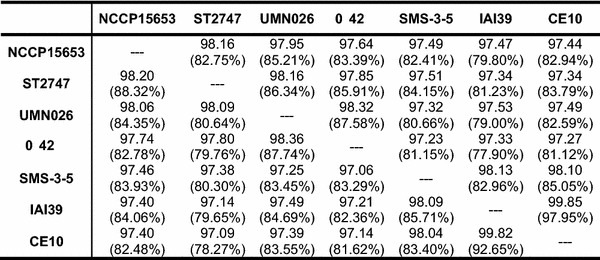
Average nucleotide identity values based on blast

Numbers in parentheses indicate the genome coverage

### Virulence genes

**Fig. 3:**
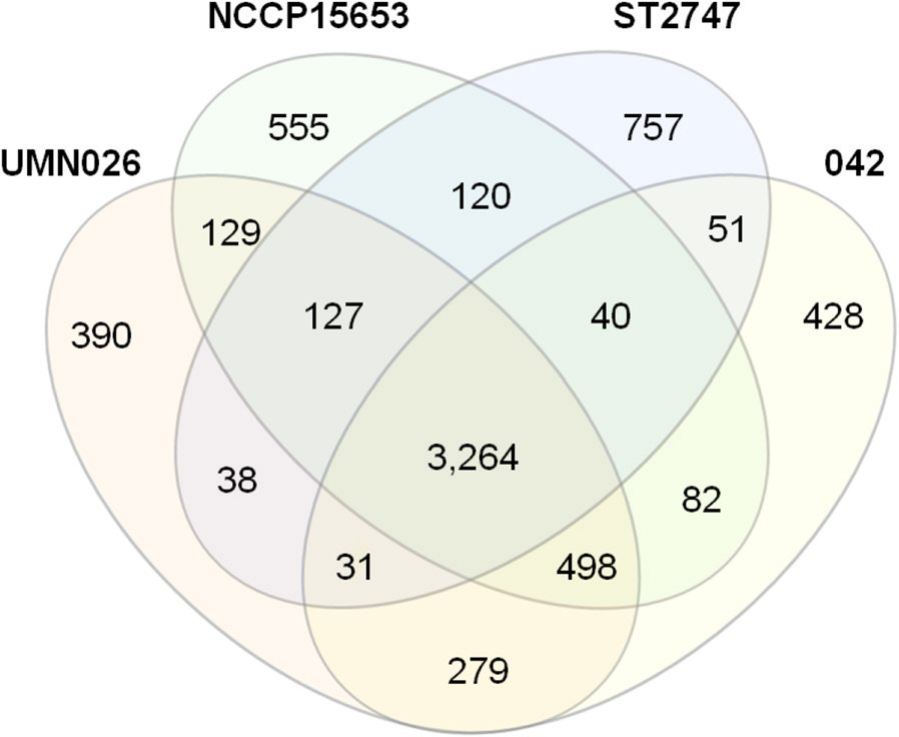
Comparison of virulence genes. The kinds of virulence genes were compared within the pathogenic strains in group D of *E. coli*. *Dark grey columns* indicate the existing genes in each genome

Dr adhesins, F1C fimbriae, P fimbriae, S fimbriae, type 1 fimbriae, immuno-evasion protein, aerobactin, enterobactin, Chu proteins, siderophore receptor, proteases, CNF-1 toxin, and hemolysin are the major virulence factors of UPEC [25]. In the genome of NCCP15653, several virulence factors for UTI were detected and shown in Fig. 3. They include genes encoding type I fimbriae, hemin uptake proteins, iron/manganese transport proteins, yersiniabactin siderophore proteins, type VI secretion proteins, and hemolysin (Fig. 3). Type I fimbriae are known to promote intracellular invasion and persistence [11], and hemolysin is known to kill the host cell by making pores to the surface [33]. Genes associated with iron-uptake are expected to make *E. coli* possible to survive in iron-deprived environments like the urinary tract [34]. In the genome of NCCP15653, genes encoding AslA and OmpA, which are virulence factors associated with invasion of NMEC, were discovered. Moreover, *kps* genes encoding proteins that form the K1 capsule were also identified in the genome of NCCP15653. The K1 capsule is known as a predominant capsular polysaccharide detected in approximately 80 % of the NMEC strains [35] and known to play important roles in invasion and survival in the host cell [36]. On the other hand, the *cnf*1 gene encoding cytotoxic necrotizing factor 1, which is a toxin of NMEC, was not present.

### Comparison with other *E. coli* strains in group D

**Fig. 4:**
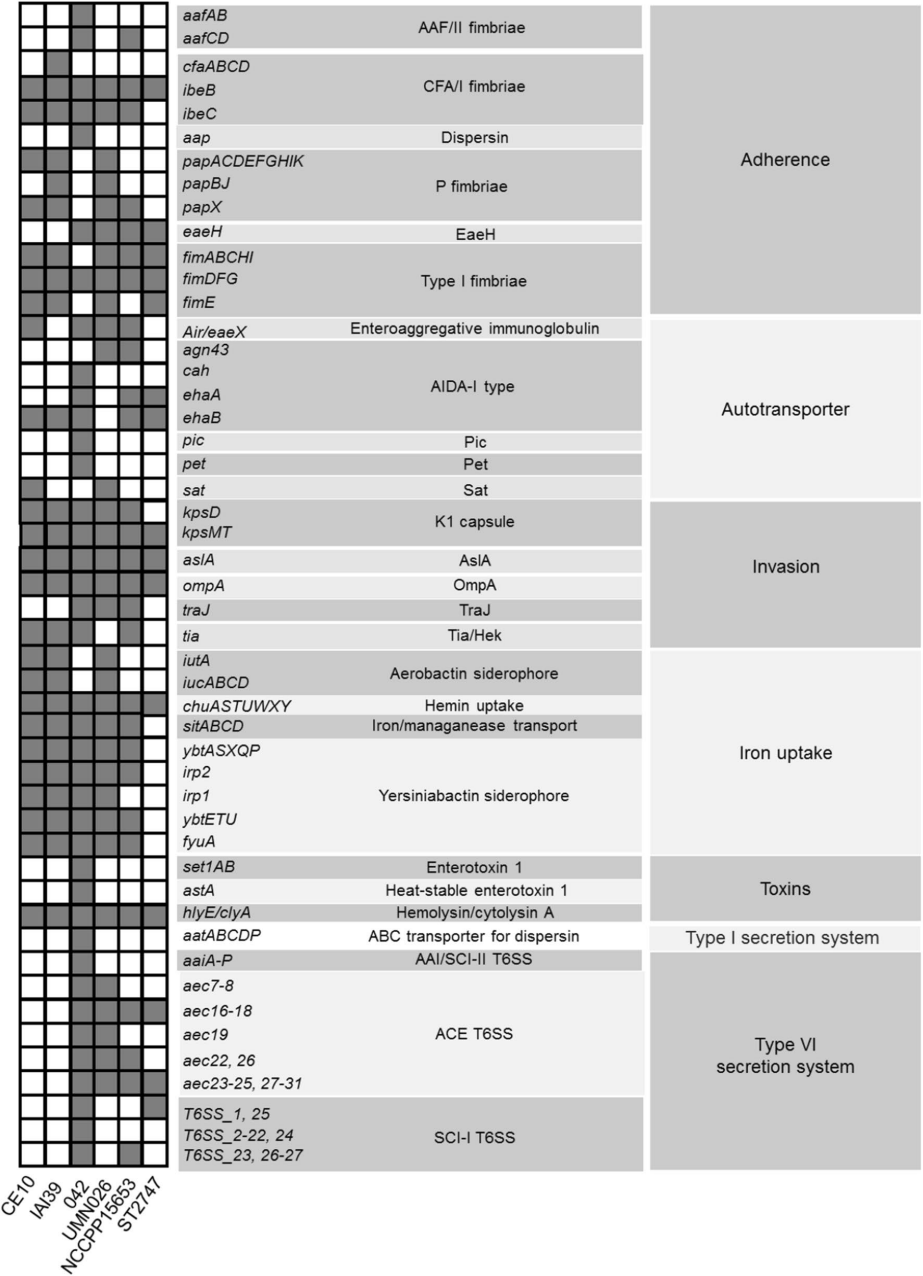
Numbers of core gene set and shared genes among strains NCCP15653, ST2747, UMN026, and 042. Gene designations are according to ref. [25]

An analysis of the core genome of four strains in group D, which were located in the same lineage with NCCP15653, inferred that they share 3264 core genes (Fig. 4). The core gene set contains genes encoding CFA/I fimbrial proteins, hemolysin E, and flagella-biosynthetic proteins and proteins as well as proteins related to general cell metabolism. Genes conserved in NCCP15653, ST2747, and UMN026, three strains that share the same common ancestor, as compared with 042, an EAEC strain outside of them, include genes encoding adhesin for cattle intestine colonization. Genes conserved in NCCP15653 and ST2747 compared with UMN026 and 042 include genes encoding entericidin and toxin-antitoxin system proteins RelB and RelE.

Comparison of the virulence genes in the pathogenic strains in group D suggests that they may be divided into three groups (Fig. 3). The first group includes CE10 and IAI39, which are NMEC and UPEC, respectively. The second has UMN026, a UPEC strain, NCCP15653, and ST2747, which was isolated from a patient with UTI, but its pathotype is not yet determined [28]. The third group contains the EAEC strain 042 alone, which has a quite different gene content compared to those in the first and second groups. Strains in the first and second groups show similar gene contents. However, in the genomes of CE10 and IAI39, *aec* (*tss*) genes encoding the type VI secretion system were not detected, and in the genome of NCCP15653, biosynthetic genes for aerobactin siderophore and P fimbriae were not present.

## Conclusions

NCCP15653 was isolated from the feces of a diarrhea patient. However, an MLST-based phylogenetic tree and ANIb values indicated that NCCP15653 belongs to the D group of *E. coli* and is a sister strain of ST2747. In addition, in the genome of NCCP15653, genes encoding UPEC-type virulence factors of were detected, and those included type I fimbriae, hemin uptake proteins, iron/manganese transport proteins, yersiniabactin siderophore proteins, type VI secretion proteins, and hemolysin. Moreover, NCCP15653 has genes associated with the invasion of NMEC, which include those for the K1 capsule and putative arylsulfatase. Genome analysis results of NCCP15653 will be useful for further research of genome dynamics in the pathogenic *E. coli* strains causing UTI.

## Availability of supporting data

This whole genome shotgun project of NCCP15653 has been deposited at GenBank under the accession ATLY00000000.
